# Supplementary material for: The interactome of intact mitochondria by cross-linking mass spectrometry provides evidence for coexisting respiratory supercomplexes
Source: Mol Cell Proteomics. 2017 Dec 8;17(2):216–32. doi: 10.1074/mcp.RA117.000470 (PMC5795388; doi:10.1074/mcp.RA117.000470)
Supplement: Supplemental Data [file supp_RA117.000470_133922_0_supp_23978_fzffwf.zip › spectra_annotation/mito_spectra_annotation/250-1-4-1-16-1.pdf]

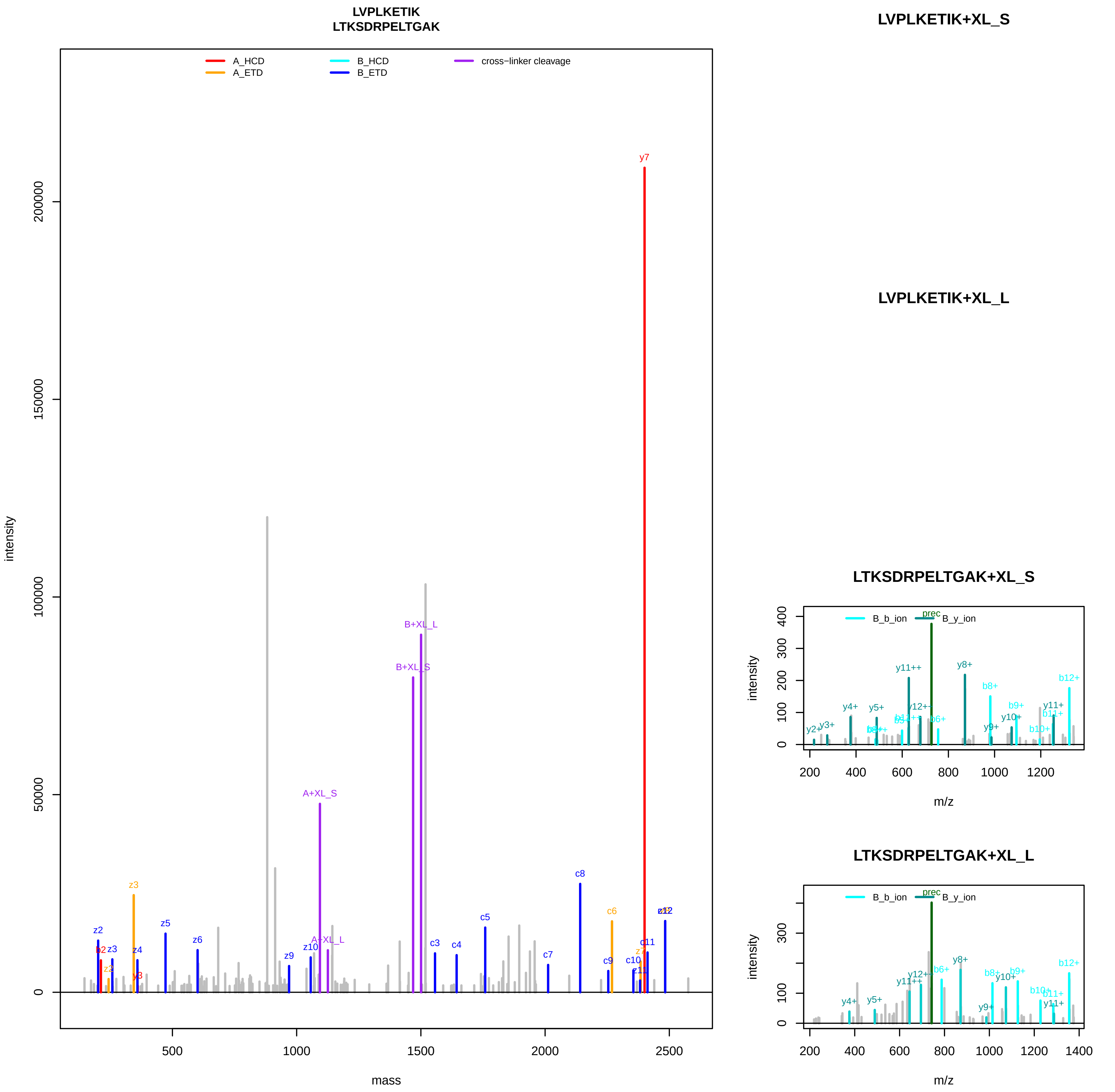

LVPLKETIK
LTKSDRPELTGAK
A_HCD
A_ETD
B_HCD
B_ETD
cross−linker cleavage
intensity
mass
0
50000
100000
150000
200000
500
1000
1500
2000
2500
y7
B+XL_L
B+XL_S
A+XL_S
A+XL_L
z2
b2
z3
z2
z3
y3
z4
z5
z6
z9
z10
c3
c4
c5
c7
c8
c9
c6
c10
z11
z7
c11
z12
LVPLKETIK+XL_S
LVPLKETIK+XL_L
LTKSDRPELTGAK+XL_S
B_b_ion
B_y_ion
prec
m/z
y11++
y8+
b8+
b12+
y12++
b6+
y4+
y5+
y2+
y3+
y9+
y10+
b9+
y11+
b11+
b10+
LTKSDRPELTGAK+XL_L
